# Gender differences in blood transfusion strategy for patients with hip fractures - a retrospective analysis

**DOI:** 10.7150/ijms.33954

**Published:** 2020-02-21

**Authors:** Rene Burchard, Alina Daginnus, Christian Soost, Jan Schmitt, Jan Adriaan Graw

**Affiliations:** 1 Department of Statistics an Econometrics, University of Siegen, Siegen, Germany.; 2 Department of Health, University of Witten/Herdecke, Witten, Germany.; 3 Department of Trauma- and Orthopaedic Surgery, Kreisklinikum Siegen, Siegen, Germany.; 4 Department of Orthopaedics and Trauma Surgery, University of Marburg, Marburg, Germany.; 5 FOM University of Applied Sciences, Essen, Germany.; 6 Department of Orthopaedics and Trauma Surgery, Lahn-Dill-Kliniken, Wetzlar, Germany.; 7 Department of Anaesthesiology and Operative Intensive Care Medicine (CCM, CVK) Charité - Universitätsmedizin Berlin, Berlin, Germany.; 8 Berlin Institute of Health (BIH), Berlin, Germany.

**Keywords:** hip fracture, blood transfusion, transfusion practice, Intensive Care Unit length of stay

## Abstract

**Background:** In the last decades, transfusion therapy with allogenic blood has progressively shifted to a more restrictive approach. The current study analyzed the transfusion practice and transfusion-associated factors in a regional trauma center over the course of five years.

**Methods:** Retrospective analysis of all patients undergoing surgery for hip fractures in a level 1 trauma center of an academic teaching hospital from 2010 to 2014 (n=650). The number of transfused packed red blood cells (PRBCs), preoperative Hb concentrations, and intensive care unit (ICU) and hospital length of stay (LOS) were analyzed. A logistic regression analysis was performed to evaluate transfusion and ICU LOS-associated risk factors. (Ethical Review Board approval: 2015-497-f-S).

**Results:** From 2010 to 2014 the average number of PRBCs transfused per patient decreased continuously despite similar preoperative Hb levels. During the same period, ICU LOS increased while hospital LOS decreased. Advanced patient age, preoperative Hb concentrations, surgical complications, and ICU LOS were associated with increased transfusion requirements. Although preoperative Hb levels were lower, females received fewer PRBCs compared to males.

**Conclusion:** Over the course of five years, a restrictive transfusion strategy was implemented within clinical practice in patients undergoing surgery for hip fractures. In parallel, a significant reduction in the hospital LOS and an increased ICU LOS was noted. Whether there is an association between increased ICU LOS and decreasing hospital LOS and whether there is a gender effect on transfusion requirements in patients with surgery for hip fractures should be subject to further research.

## Introduction

A transfusion of packed red blood cells of volunteer donors is a life-saving therapy for patients with anemia or major blood loss. Besides cardiac output and pulmonary oxygen uptake, the number of red blood cells in the circulation determines the blood oxygen carrying capacity and secures vital organ perfusion 1. Infectious, immunologic, or biochemical complications are seldom but relevant side effects of allogenic blood transfusions that can occur despite the currently high safety standards 2,3. In recent decades it has become evident that in the perioperative setting, it is outcome-beneficial to rather accept a certain degree of anemia instead of trying to maintain normal values of hemoglobin concentration with allogenic blood transfusions 4. However, the exact threshold to indicate a blood transfusion remains controversial and depends among others on the presenting disease, patient specific comorbidities, perioperative complications, and resources 5-8.

In 2011, the FOCUS-trail revealed that a higher threshold for blood transfusions (Hb-concentration of 10 g/dl) was not superior in reducing mortality in patients who had undergone surgery for hip fractures compared to a restrictive approach with a lower threshold (Hb-concentration of 8 g/dl) 9. Moreover, in elderly patients with hip surgery and a high cardiovascular risk, the liberal transfusion strategy was not associated with a reduced in-hospital morbidity compared to the restrictive approach 9. These findings were concordant with previously obtained data in critically ill patients and from smaller trials 10.

The aim of this study was to analyze how transfusion practice has changed during the years after the paradigm change on transfusion thresholds in patients that underwent surgery for hip fractures in a regional trauma center. In addition, variables and complications associated with an allogenic blood transfusion in patients with a hip fracture were analyzed.

## Methods

This retrospective observational single-center study was performed in a 595-bed academic teaching hospital. The Department of Trauma and Orthopedic Surgery serves as one of two level one regional trauma referral centers for an area with more than 277,000 inhabitants. The study included all patients admitted with a bony fracture of the thigh requiring surgical treatment from 2010 to 2014. Fractures consisted of medial and lateral, as well as pertrochanteric and subtrochanteric femur fractures and surgical treatment included total hip arthroplasty, a femoral hemiarthroplasty of the hip, or an intramedullary femur nail. Anemia was defined as a hemoglobin-concentration of less than 12 g/dl in females and less than 13 g/dl in males 6.

Patient records in the Department of Trauma an Orthopedic Surgery are kept electronically with a Patient Data Management System (MCC Meierhofer®, Meierhofer AG, Munich, Germany). Demographic patient data, type of injury and surgical treatment, preoperative blood counts, intensive care unit and hospital length of stay, and data on transfusion requirements during the entire hospital length of stay were collected retrospectively. A comorbidity score was defined according to preoperative comorbidities such as dementia, congestive heart failure, stroke, and ataxia. Presence of significant comorbidities was considered when ≥1 of the mentioned comorbidities was present. Thrombembolic events were defined as pulmonary embolism, deep vein thromboembolism, ischemic stroke, and myocardial infarction. Death, thrombembolic events, perioperative pneumonia and urinary tract infection were considered as systemic complications, whereas wound infections and need for revision surgery were considered as surgical complications. The Medical Ethics Committee of the Medical Council Westphalia-Lippe approved this study (number of ethical approval: 2015-497-f-S). Informed consent was waived due to the retrospective and observational nature of the study.

Results are expressed in absolute numbers and frequencies (%) or mean and standard deviation (SD) unless indicated otherwise. The Shapiro-Wilk test was used to assess normal distribution of measured variables. To describe changes over time, the patient cohort of 2010 served as reference group. Continuous variables of two independent groups were compared using non-parametric Wilcoxon-Mann-Whitney test. Statistical comparison between more than two groups was assessed by Kruskal-Wallis test with an uncorrected Dunn´s post-hoc test for pairwise comparison. Frequencies were tested by the (exact) Chi-square-test in contingency tables. Ordinal regression modeling was conducted to evaluate associations between socio-demographic and surgical variables and the transfusion requirements. Variables introduced in the models included: patient age, sex, perioperative cardiovascular complications, surgical and local complications, surgical treatment, and intensive care unit and hospital length of stay. A two-tailed P-value <0.05 was considered statistically significant. Data were analyzed using GraphPad Prism software (GraphPad Software Inc., La Jolla, CA) and statistical software package SPSS® Version 24 (IBM, Armonk, North Castle, New York, USA). Regression modeling was conducted using R (www.r-project.org).

## Results

**Figure 1:**
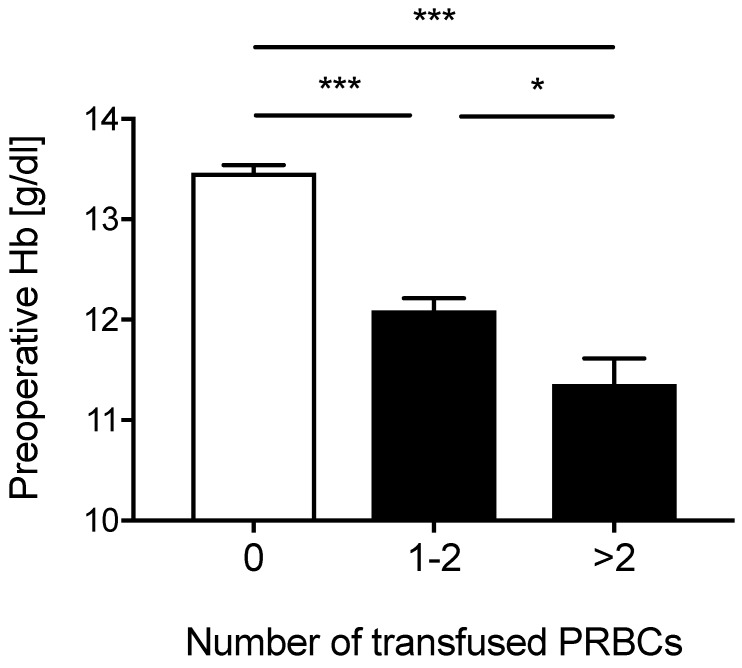
** Preoperative Hemoglobin concentrations.** Preoperative hemoglobin (Hb) concentrations according to the number of transfused packed red blood cells (PRBCs) for all patients. Data represent means ± SE, *P<0.05, ***P<0.001 vs. 0, Kruskal-Wallis test with a Dunn´s post-hoc test for pairwise comparison.

Table 1 shows epidemiologic data of all 650 patients (28.5% male) that were included in the analysis. A third of these patients (n=220, 33.8%) received an allogenic blood transfusion. One-hundred seventy-six (27.2%) of the patients with blood transfusions received one or two units of packed red blood cells and 44 (6.8 %) patients received more than two units of allogenic blood. Patients who received a blood transfusion had a lower preoperative hemoglobin-concentration compared to patients who were not transfused (Figure 1). In addition, preoperative hemoglobin levels in patients who received one to two units of packed red blood cells were greater compared to patients that received more than two units of packed red blood cells (Figure 1).

**Figure 2:**
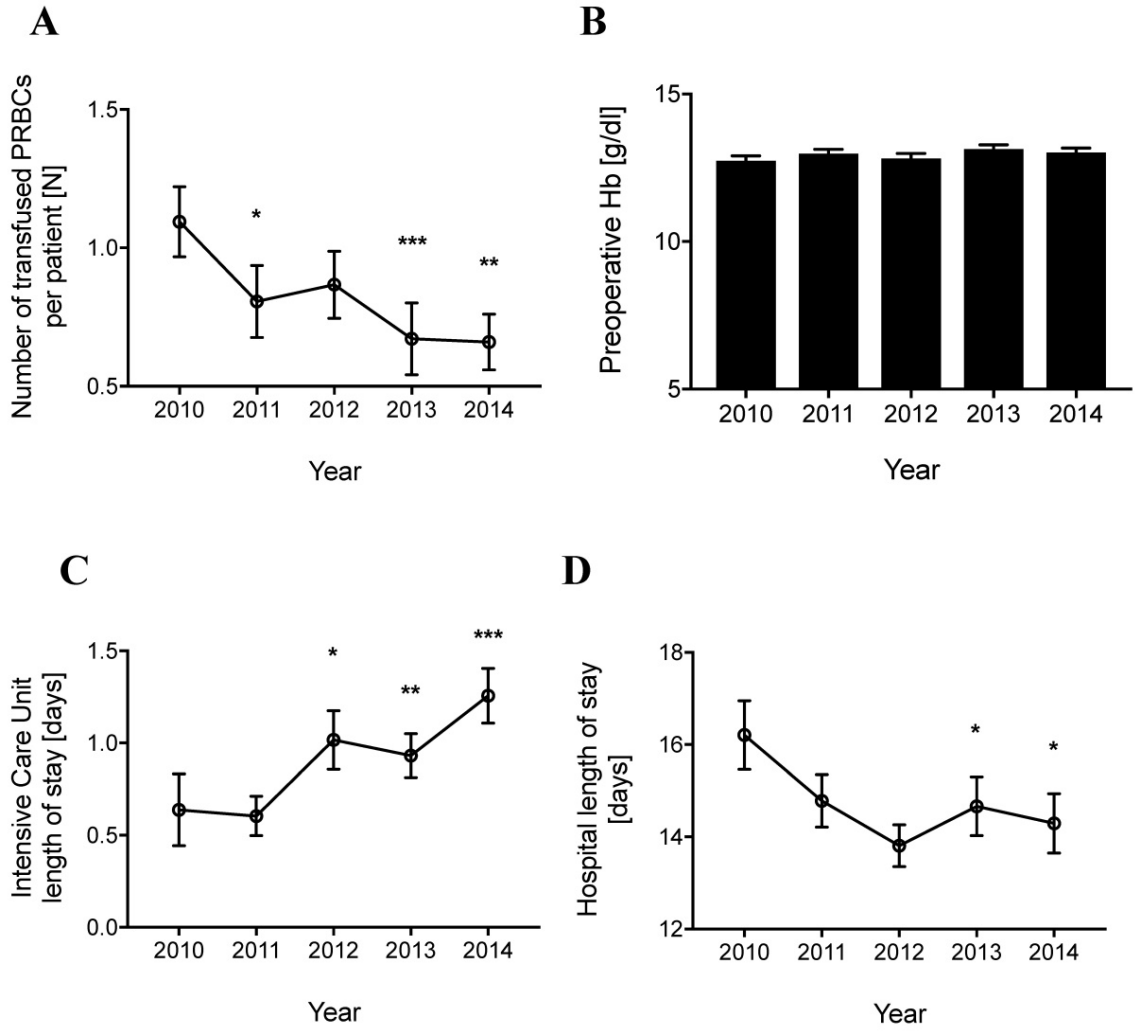
** Number of transfused packed red blood cells, the preoperative hemoglobin concentration, and the Intensive Care Unit and hospital length of stay in the years from 2010 to 2014.** (A) Number of transfused packed red blood cells (PRBCs) per patient per year (B) Average preoperative hemoglobin (Hb) concentration per year (C) Intensive Care Unit (ICU) length of stay (LOS) per patient per year (D) Hospital LOS per patient per year. Data represent means ± SEM, *P<0.05, **P<0.01, ***P<0.001 vs. group of 2010, Kruskal-Wallis test with a Dunn´s post-hoc test for pairwise comparison.

To evaluate the transfusion practice over time, the number of transfused packed red blood cells per patient per year was analyzed. Figure 2A demonstrates that the number of transfused of packed red blood cells decreased continuously from 1.10±0.13 units per patient in 2010 to 0.65±0.10 units per patient in 2014 (p<0.01). Furthermore, the average preoperative hemoglobin-concentration in all patients was similar for each year (Figure 2B). In addition, the preoperative hemoglobin-concentration in patients that received no blood transfusion, one or two units of packed red blood cells, or more than two units of packed red blood cells did not differ among the years.

The intensive care unit length of stay and the hospital length of stay were analyzed for the same period. The average intensive care unit length of stay per patient increased continuously from 0.64±0.20 days in 2010 to 1.26±0.15 days in 2014 (Figure 2C). In contrast, the average hospital length of stay per patient decreased from 16.21±0.74 days in 2010 to 14.29±0.65 days in 2014 (Figure 2D). Taken together, in the five-year period from 2010 to 2014 a decrease in blood transfusion requirements and hospital length of stay and an increase of the intensive care unit length of stay was detected in patients with hip fractures admitted to this regional trauma center.

An ordinal regression analysis was performed to detect variables that were associated with the transfusion of packed red blood cells in this patient cohort (Table 2). Besides advanced patient age and low preoperative Hb-levels, surgical complications and increased intensive care unit length of stay were associated with increased transfusion requirements. However, perioperative systemic complications and a set of comorbidities were not associated with increased transfusion requirements. Furthermore, female sex was associated with a lower number of blood transfusions compared to male sex.

**Figure 3:**
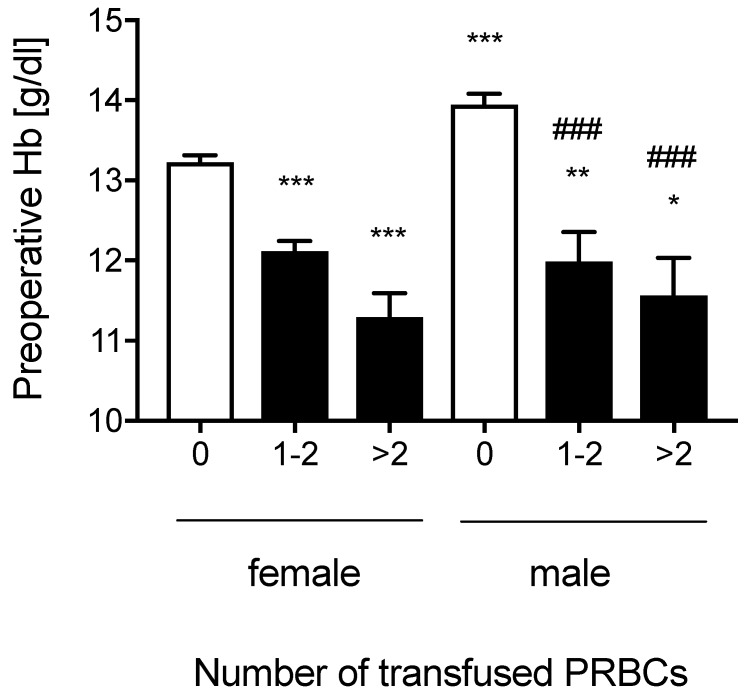
** Preoperative Hemoglobin concentrations in males and females.** Preoperative hemoglobin (Hb) concentrations according to the number of transfused packed red blood cells (PRBCs) separated for males and females. Data represent means ± SEM, *P<0.01, **P<0.01, ***P<0.001 vs. females 0; ###P<0.001 vs. males 0, Kruskal-Wallis test with a Dunn´s post-hoc test for pairwise comparison.

Women had lower preoperative hemoglobin-concentrations compared to men (Hb_female_: 12.74±0.08 g/dl vs. Hb_male_: 13.48±0.14 g/dl, p<0.001). There was no gender difference in the proportion of patients with preoperative anemia (Anemia-frequency_females_: 29.5% vs. Anemia-frequency_males_: 34.1%, p=0.260). In patients that received one to two units or more than two units of packed red blood cells, preoperative hemoglobin-concentrations measured in females did not differ from preoperative hemoglobin-concentrations measured in males (Figure 3).

## Discussion

The current evidence of a restrictive transfusion strategy in patients with a hip fracture was implemented into the clinical practice of a regional trauma center in the years after publication of the FOCUS-trail 9. Parallel to a decrease of the hospital length of stay, the intensive care unit length of stay increased over the years. Furthermore, female sex was associated with decreased transfusion requirements.

Almost a third of patients were anemic before surgery. Anemia is a frequent finding in geriatric patients and data from Germany report a prevalence of more than 50% in patients above the age of 70 11,12. Lower preoperative hemoglobin-concentrations were associated with a higher rate of blood transfusions. Previous data suggest that preoperative hemoglobin-concentrations are not associated with the hospital length of stay while lower postoperative hemoglobin-concentrations correlate with a longer hospital length of stay 13. Because the preoperative hemoglobin-concentrations did not differ over the years in the studied population and neither the surgical team nor the major surgical techniques have changed during the study period, implementation of a more restrictive transfusion strategy can be one of the factors that might explain the decrease of blood transfusions from 2010 to 2014 in patients with hip surgery.

Interestingly, during the same observation period an increase in the average intensive care unit length of stay with a concomitant decrease in the hospital length of stay was noted. One might speculate that patients with lower postoperative hemoglobin levels based on a more restrictive transfusion approach, might receive an extended and closer clinical monitoring during the initial postoperative period. However, the nature of this study does not infer that these effects might be associated with the described change in transfusion practice.

Besides age, preoperative hemoglobin-concentrations are a known and relevant factor for increased transfusion requirements in patients with hip fractures 14. Generally, females have a higher prevalence of anemia 15. Likewise, in the studied patient population the preoperative hemoglobin-concentration was significantly higher in males compared to females. In men anemia prevalence increases with age and in geriatric patients it does not differ from anemia prevalence in females any more 15. In patients that were transfused, preoperative hemoglobin-concentrations did not differ between genders. However, females were transfused less frequently than men, an association that was also supported by the binary logistic regression analysis. These data are in line with findings of another recent study that included patients aged ≥70 years with hip fractures 14. Therefore it would be interesting whether this gender effect persists in a larger prospective trial. Currently, the gender is not considered as a factor that triggers indications for a blood transfusion and no national or international recommendation on indications for blood transfusions considers gender as a relevant factor to trigger transfusion therapy 4, 16-18.

The current study is limited by its retrospective and single center design. Furthermore, it is likely that other factors besides the increased awareness on transfusion strategies in patients for urgent hip surgery after the FOCUS-trail might have led to a reduction in transfusion requirements over the studied years. However, the national recommendations on indications and triggers for a transfusion with allogenic packed red blood cells were not changed during and after that period 18. In addition, unknown cofounders might have introduced a bias into the multivariate regression analysis.

Taken together this study indicated that a restrictive approach for the transfusion of allogenic packed red blood cells has been introduced into clinical practice for patients with urgent hip surgery in a regional trauma center in the time frame when the FOCUS-trial was published. Over five years, a significant reduction in the hospital length of stay with an increased length of stay on the intensive care unit was recognized. Whether there is an association between an increased intensive care unit length of stay and a decreasing hospital length of stay and whether there is a gender effect on transfusion requirements in patients with surgery for hip fractures should be subject to further research.

## Figures and Tables

**Table 1:** Characteristics of patients admitted with a hip fracture

Patient characteristics	Patients without blood transfusion (n=430)	Patients with blood transfusion (n=220)	P-value
Age, years	77.58 ± 12.80	82.49 ± 8.74	<0.001
Gender, female, n (%)	287 (66.7)	178 (80.9)	<0.001
Preoperative Hb, g/dl	13.47 ± 1.54	11.95 ± 1.69	<0.001
**Treatment**			
Intracapsular hip fractures, n (%)	199 (46.3)	99 (45.0)	0.803
Extracapsular hip fractures, n (%)	231 (53.7)	121 (55.0)	
**Adverse Events**			
Death, n (%)	23 (5.3)	13 (5.9)	0.768
Thrombembolic events, n (%)	5 (1.2)	4 (1.8)	0.496
Wound revision, n (%)	8 (1.9)	12 (5.5)	0.016
Wound infection, n (%)	8 (1.9)	10 (4.5)	0.074
**Hospitalization**			
ICU > 1 day, n (%)	122 (28.4)	101 (45.9)	<0.001
ICU LOS, days	0.7 ± 1.39	1.31. ± 2.10	<0.001
Hospital LOS, days	13.73 ± 6.07	16.66 ± 8.51	<0.001

Hb=hemoglobin concentration, ICU = Intensive Care Unit, LOS = Length of stay.

**Table 2:** Ordinal regression analysis for factors associated with blood transfusions

Blood transfusion	Estimate	P-value	OR	CI
Age	0.019	0.013	1.019	(1.006 - 1.033)
Gender (female)	-0.365	0.038	0.694	(0.500 - 0.924)
Preoperative Hb-concentration	-0.419	<0.001	0.657	(0.605 - 0.702)
Comorbidities	0.061	0.697	1.063	(0.818 - 1.396)
Surgical technique (intramedullary femur nail)	-0.034	0.808	0.966	(0.761 - 1.225)
Systemic complications	-0.324	0.280	0.723	(0.413 - 1.186)
Surgical complications	0.775	0.008	2.170	(1.208 - 3.998)
ICU LOS	0.167	<0.001	1.182	(1.093 - 1.269)

Hb=hemoglobin concentration, ICU = Intensive Care Unit, LOS = Length of stay.
